# Recent Advances in Zirconium-89 Chelator Development

**DOI:** 10.3390/molecules23030638

**Published:** 2018-03-12

**Authors:** Nikunj B. Bhatt, Darpan N. Pandya, Thaddeus J. Wadas

**Affiliations:** Department of Cancer Biology, Wake Forest University Health Sciences, Winston-Salem, NC 27157, USA; nbhatt@wakehealth.edu (N.B.B.); dapandya@wakehealth.edu (D.N.P.)

**Keywords:** zirconium-89, chelator, positron emission tomography

## Abstract

The interest in zirconium-89 (^89^Zr) as a positron-emitting radionuclide has grown considerably over the last decade due to its standardized production, long half-life of 78.2 h, favorable decay characteristics for positron emission tomography (PET) imaging and its successful use in a variety of clinical and preclinical applications. However, to be utilized effectively in PET applications it must be stably bound to a targeting ligand, and the most successfully used ^89^Zr chelator is desferrioxamine B (DFO), which is commercially available as the iron chelator Desferal^®^. Despite the prevalence of DFO in ^89^Zr-immuno-PET applications, the development of new ligands for this radiometal is an active area of research. This review focuses on recent advances in zirconium-89 chelation chemistry and will highlight the rapidly expanding ligand classes that are under investigation as DFO alternatives.

## 1. Introduction

Over the last four decades, molecular imaging has had a transformative effect on the way research is conducted in academia, industry and on how medical care is managed in the clinic [1,2,3,4,5,6,7,8]. Of the modalities available to preclinical researchers and clinicians, the popularity of the nuclear medicine technique positron emission tomography (PET) has surged since it provides physiological data relating to disease pathophysiology, receptor expression levels, enzyme activity and cellular metabolism non-invasively and quantitatively [9,10,11,12]. PET imaging relies upon the unique decay characteristics of PET radionuclides, which decay by positron emission, and are chemically attached to ligands designed to probe biochemical phenomena in vivo [13,14]. As the radionuclide decays, it ejects a positron from its nucleus, which after travelling a short distance, undergoes a process called annihilation with an electron to release two 511 keV *γ* rays 180° apart. These coincident gamma rays have sufficient energy to escape the organism and can be detected by the PET scanner. Computer-based algorithms then convert the signal data into an image that reveals the distribution of the radiotracer within the organism. Historically, PET isotopes such as ^18^F, ^15^O, ^13^N, ^11^C and ^68^Ga; which have relatively short half-lives, were developed for use with small molecules or peptides that demonstrated rapid target tissue accumulation and clearance, and facilitated the imaging of physiological processes within the first 24 h of radiopharmaceutical injection [15]. However, researchers engaged in the development of monoclonal antibodies, which represent one of the fastest growing therapeutic groups, were unable to take full advantage of PET as a molecular imaging technique. The aforementioned radionuclides had half-lives incompatible with the biological half-life of an antibody, and made imaging their biodistribution days after injection extremely difficult. While several PET radionuclides such as ^64^Cu, ^86^Y and ^124^I have been used in the development of mAb-based radiopharmaceuticals, they possess undesirable physical, chemical or radioactive properties that have minimized their use [15,16,17]. For example, ^64^Cu and ^86^Y have half-lives, which are incompatible with the slow pharmacokinetics displayed by an antibody in vivo. Furthermore, dehalogenation of ^124^I-radiolabeled antibodies in vivo coupled with the low resolution images they produce have left the molecular imaging community with little enthusiasm to apply this PET radionuclide for the diagnostic imaging of disease. However, the introduction of zirconium-89 (^89^Zr) more than three decades ago has reinvigorated this rapidly expanding area of research known as immuno-PET [18,19]. Its impact on antibody and nanoparticle development, clinical trials and precision medicine strategies has been reviewed extensively [14,15,16,20,21,22,23,24,25,26,27,28,29,30,31,32,33,34,35,36,37,38,39,40,41,42,43,44,45,46].

## 2. Zirconium Chemistry and the Production of Zirconium-89

Zirconium, a second row transition metal, was first isolated by Berzelius in 1824 [47], and since that time numerous inorganic and organometallic complexes of Zr have been described with zircon (ZrSiO_4_), being its most widely recognized inorganic form [48,49,50,51]. Zirconium can exist in several oxidation states including Zr(II), Zr(III) and Zr(IV), which is its preferred oxidation state [48]. Zirconium (II) complexes are known, but they typically require p-donor ligands to enhance stability even under inert atmosphere conditions, and even fewer reports describing the Zirconium (III) oxidation state exist. A significant portion of knowledge regarding this element’s reactivity has been extrapolated from hafnium (Hf) chemistry since their atomic and ionic properties yield similar chemistries with a variety of ligands, and much of what is known about zirconium coordination chemistry has been discovered in the context of solid-state material or catalysis development [52,53]. While research in these areas has provided numerous societal benefits including heat and corrosion resistant coatings; fracture resistant ceramics; and the development of catalysts that play a role in the petroleum, plastics, and pharmaceutical industries, it has been difficult to translate this knowledge into the research fields of radiochemistry and molecular imaging. The requirements of zirconium complexes in the latter arenas are completely different from the former branches of scientific inquiry. For example, typical catalytic applications require a non-aqueous environment and a zirconium complex with labile ligands [54,55,56,57,58,59,60,61,62], but for molecular imaging applications, zirconium complexes must be extremely hydrophilic and inert to ligand substitution or loss [14]. Further complicating the exploration of zirconium radioisotopes in molecular imaging is its complex aqueous chemistry [14,16,63,64,65]. Currently, experimental evidence indicates that due to its high charge and small radius, hydrated Zr(IV) exists as multiple monomeric and polynuclear µ-oxy- and µ-hydroxy-bridged species in solution at low pH. The nature and abundance of these species can change depending upon pH, while an increasing solution pH favors the formation and precipitation of zirconium hydroxide species. This has made the accurate determination of stability constants with various chelating ligands very difficult. 

**Figure 1:**
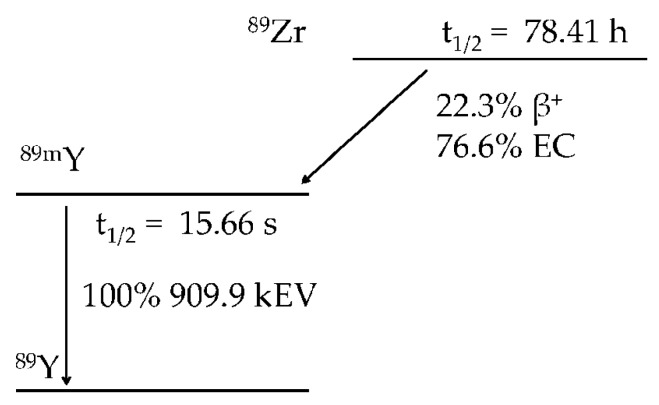
Zirconium-89 decay scheme. Zirconium-89 decays by positron emission and electron capture to metastable yttrium-89. Metastable yttrium-89 decays by gamma emission to stable yttrium-89.

While several isotopes of Zr including ^86^Zr (*t*_1/2_: 17 h, *γ* 100%, *E_γ_* = 241 keV), ^88^Zr (*t*_1/2_: 85 d, *γ* 100%, *E_γ_* = 390 keV), and ^89^Zr (*t*_1/2_: 78.4 h, *β*+ 22.8%, *E β*+_max_ = 901 keV; 901 keV, EC 77%, *E_γ_* = 909 keV) can be produced on a cyclotron [66,67], ^89^Zr has received the most attention for radiopharmaceutical development because of its favorable nuclear decay properties that make it useful in the labeling of antibodies for immuno-PET applications (Figure 1) [68,69,70]. The availability of carrier-free ^89^Zr as either zirconium-89 oxalate ([^89^Zr]Zr(ox)_2_) or zirconium-89 chloride ([^89^Zr]Zr Cl_4_) is essential to the development of effective immuno-PET agents. Link et al. were the first to produce ^89^Zr by a (p,n) reaction by bombarding ^89^Y foil with 13 MeV protons [18]. After irradiation, ^89^Zr was purified by a double extraction protocol followed by anion exchange and elution with oxalic acid to afford ^89^Zr (as [^89^Zr]Zr(ox)_2_) in an 80% yield and with a purity greater than 99%. Although incremental improvements were made in the production and purification of ^89^Zr soon after that [71,72] a major advance in ^89^Zr production was reported by Meijs and coworkers, who were able to produce ^89^Zr using the (p,n) reaction and 14 MeV protons produced on a Philips AVF cyclotron [73]. After oxidation of the target material, other metal impurities were removed by anion exchange chromatography using a hydroxamate-modified resin, which was chosen because of this coordinating unit’s ability to form complexes with ^89^Zr(IV) under highly acidic conditions. This allowed the ^89^Zr to be retained within the column while the other metal impurities were removed under low pH conditions. The purified ^89^Zr was then eluted in 95% yield using 1 M oxalic acid, which was removed by sublimation under vacuum. Using this method the authors were able to prepare highly pure [^89^Zr]Zr(ox)_2_ for subsequent radiochemical applications, which were later incorporated into comprehensive procedures for preparing ^89^Zr-labeled antibodies [74]. Later, Holland et al. demonstrated how to maximize recovery of isotopically pure ^89^Zr with an achievable molar activity of more than 1000 Ci/nmol by examining ^89^Zr production as a function of cyclotron irradiation time, and purification of the target material as a function of the concentration-dependent loading efficiencies of the hydroxamate resin [75]. Additionally, the authors described improved processes for making [^89^Zr]ZrCl_4_. These findings were instrumental in automating [^89^Zr]Zr(ox)_2_ production and seized upon by other research groups, who have endeavored to increase the availability of high molar activity [^89^Zr]Zr(ox)_2_ and [^89^Zr]ZrCl_4_ [67,76,77,78,79,80,81,82]. 

## 3. The Rationale for New Zirconium-89 Chelation Strategies

**Figure 2:**
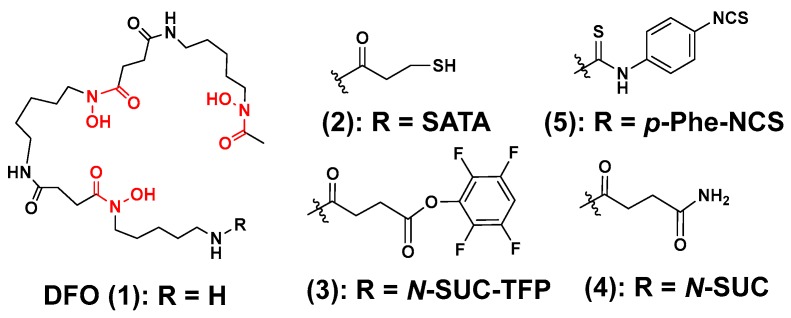
DFO-based bifunctional chelators for ^89^Zr. The coordinating units are depicted in red font.

**Figure 3:**
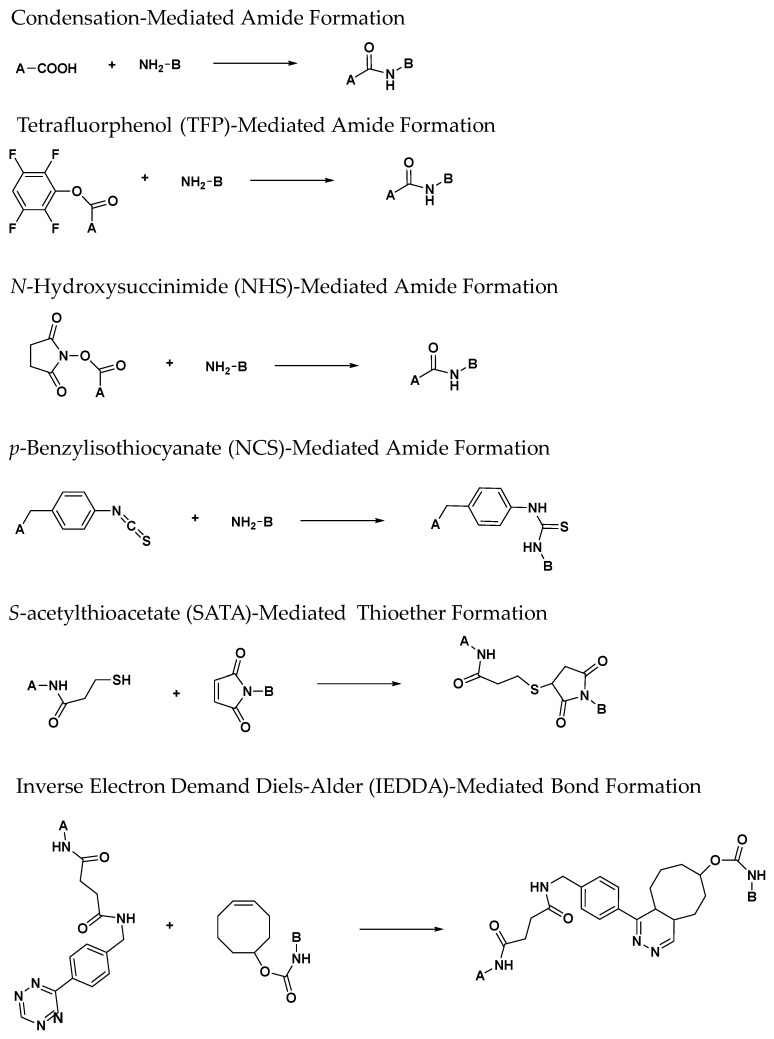
Selected bioconjugation reactions used to link ^89^Zr-bifunctional chelators A with targeting ligands B that are described in this text. For clarity, leaving groups and reaction conditions are not shown.

Medical researchers have always found inspiration in nature when developing new treatments to combat disease. In a similar manner, chemists have developed ligands for ^89^Zr chelation, which have been inspired by siderophores or the chelating agents produced by bacteria and fungi to sequester metal ions from the environment [83,84,85]. The desferrioxamines are a class of iron (III) binding-siderophores that are synthesized from the amino acids lysine and ornithine and contain a tris-hydroxamate coordination motif [84,85,86]. Given Zr’s preference for hard, anionic donor groups, and its ability to form complexes with mono-hydroxamates, it was reasonable to assume these types of iron-binding ligands would be valuable in ^89^Zr radiochemistry. This rationale led Meijs et al. to perform the first evaluation of desferrioxamine B (DFO; **1**) as a ^89^Zr chelator, which was observed to be highly stable in human serum (Figure 2) [87]. Since that time, many derivatives have been prepared to facilitate bioconjugation to antibodies using the strategies depicted in Figure 3 [88,89]. Initially, the derivative, *N*-(*S*-acetyl)mercatopacetyldesferrioxamine B (SATA-DFO; **2**) was prepared for mAb coupling using a strategy that involved reacting SATA-DFO with maleimide-modified lysine side chains on the mAb surface to yield a thioether linkage between the DFO chelator and targeting mAb [90]. However, due to instability at physiological pH, this method was abandoned. Later, reacting the activated 2,3,5,6-tetrafluorphenol ester-modified DFO (**3**) with the primary amine side chains of solvent accessible lysine residues located on the mAb surface, Verel et al. were able to conjugate **3** to the U36 mAb through a succinamide linkage (**4**) [74]. Using this conjugate the authors then prepared [^89^Zr]Zr-DFO-*N*-SUC-U36 mAb, and evaluated it in a murine model bearing xenografts derived from the HNX-OE, human head and neck carcinoma cell line. Tumor-to-non-target background contrast improved over the time course of the study with tumors being easily visualized at 72 h post-injection. Acute biodistribution studies demonstrated that radioactivity retention in tissue was consistent with a ^89^Zr-labeled mAb. Despite the promising results obtained, the cumbersome preparation strategy, which involved chelation of Fe(III) and its EDTA-mediated removal from DFO before ^89^Zr radiochemistry could be performed, was also abandoned due to its complexity. 

Several years later, Perk et al. described the new bifunctional chelator *p*-isothiocyanato-benzyl-desferrioxamine B (DFO-*p*-Phe-NCS; **5**) as superior to the TFP-*N*-SUC-DFO and SATA-DFO BFC analogs [91]. The underlying conjugation strategy relied upon the stable formation of a thiourea linkage between the antibody and the chelator, and the one-step coupling process was complete within 60 min when the ligand and the mAb were reacted at 37 °C under highly basic conditions. To demonstrate utility, the authors prepared DFO-*p*-Phe-NCS-U36 mAb with an achievable chelator-to-mAb ratio of 1.5. They then compared the radiochemistry of this conjugate with that of DFO-*N*-SUC-U36, which was prepared using the seven step, TFP method. Radiochemical studies demonstrated comparable radiochemical yields were achieved for both conjugates allowing the authors to conclude the thiourea bond did not interfere with the radiochemistry of the conjugate. Despite facile radiochemistry, the authors did note that the NCS-derived conjugates were less stable in solution. Although no radiolysis experiments were conducted, this instability was attributed to in situ radiolysis, which could be mitigated by formulating the ^89^Zr-radiopharmaceutical in serum. In an effort to further compare conjugation strategies, the biodistribution of [^89^Zr]Zr-DFO-*p*-Phe-NCS-U36 and the [^89^Zr]Zr-DFO-*N*-SUC-U36 were compared in mice bearing FaDu human xenografts that were derived from the human pharynx squamous carcinoma cell line. In vivo results were similar for both radiopharmaceuticals indicating that the different conjugation strategies did not alter the biodistribution in this murine model, and these results were corroborated by small animal PET/CT imaging. After 72 h, the subcutaneous xenografts were clearly visible with excellent image contrast. To further demonstrate the applicability of this approach, the authors also prepared [^89^Zr]Zr-DFO-*p*-Phe-NCS-rituximab and evaluated it in a nude mouse model bearing tumors that were derived from the A431 human squamous carcinoma cell line. Results in this model were similar to those obtained using the U36 mAb and FaDu animal model. Since these initial reports, this strategy has been universally adopted for preclinical research and clinical trials because of its advantages, which include facile reaction chemistry and its adaptability to good manufacturing compliant processes (cGMP).

Despite extensive use of DFO-*p*-Phe-NCS in ^89^Zr-Immuno-PET applications, questions regarding [^89^Zr]Zr-DFO instability during the extended circulation of the radiolabeled mAb in vivo have appeared in the literature [14,16,27,92,93]. Current scientific consensus suggests that the unsaturated coordination sphere of [^89^Zr]Zr-DFO in combination with perturbation by endogenous serum proteins during the extended circulation of the mAb-based radiopharmaceutical is responsible for this observed instability, ^89^Zr transchelation, and eventual deposition into the phosphate-rich hydroxylapatite matrix found in bone [94,95,96]. Unfortunately, the presumed instability of the [^89^Zr]Zr-DFO complex has complicated the preclinical evaluation of therapeutic antibodies and also may complicate the interpretation of clinical trial results designed to improve clinical care [97]. These reports of instability fueled a desire within the research community to understand the requirements needed to form a stable ^89^Zr complex and generate new ligands that enhance the stability of the resulting ^89^Zr complex [98]. The remainder of this review will discuss recent progress in ^89^Zr chelator research and highlight the major coordinating units being incorporated into their design [99,100].

### 3.1. Zirconium-89 Chelators Containing Hydroxamate Coordinating Units

**Figure 4:**
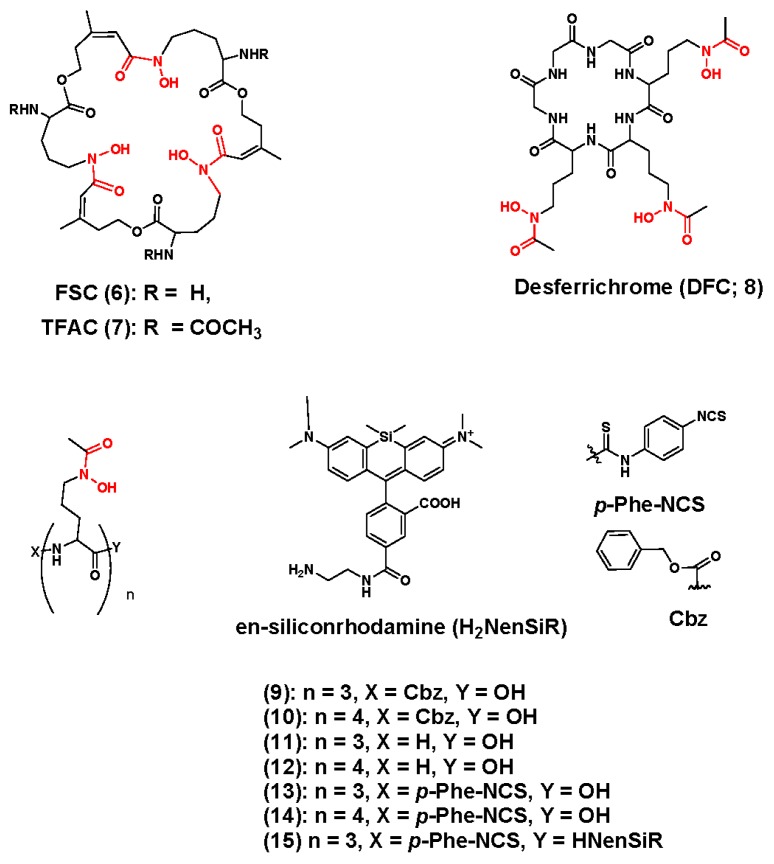
Hydroxamate-containing Zirconium-89 chelators inspired by the siderophores fusarine C and desferrichrome. The coordinating units are depicted in red font.

In addition to the desferrioxamines, additional siderophores have stimulated the creativity of molecular imaging scientists. For example, Zhai et al. examined fusarine C (FSC; **6**), which was previously evaluated as a ^68^Ga chelator, and its triacetylated analog TFAC (**7**) as ^89^Zr chelators [101]. They are depicted in Figure 4. The design benefits of these ligands include the three hydroaxamtae groups for ^89^Zr coordination, the cyclic structure to improve stability and three primary amine groups, which are amenable to a variety of bioconjugation strategies and also offer the possibility of multivalent targeting. Initially the authors studied TFAC radiochemistry and observed excellent complexation kinetics. Within 90 min the hydrophilic complex, [^89^Zr]Zr-TFAC could be prepared from [^89^Zr]Zr(ox)_2_ with a molar activity of 25 GBq/µmol. Interestingly, this research group also examined the preparation of ^Nat^Zr-TFAC using ^Nat^ZrCl_4_. Analysis of their results led the research team to support the initial claims of Holland et al., who stated that [^89^Zr]ZrCl_4_ might be superior to [^89^Zr]Zr(ox)_2_ [75]. In vitro, [^89^Zr]Zr-TFAC demonstrated greater stability against EDTA challenge compared to [^89^Zr]Zr-DFO. Additionally, biodistribution and small animal PET/CT studies of [^89^Zr]Zr-TFAC revealed rapid blood clearance with predominate renal excretion and minimal bone uptake suggesting that the [^89^Zr]Zr-TFAC complex was stable over the short time course of the study. In additional studies the authors prepared [^89^Zr]Zr-FSC-RGD and [^89^Zr]Zr-DFO-RGD. They then evaluated each radiopharmaceutical using receptor binding studies and the M21 (α_v_β_3_^+^) and M21L (α_v_β_3_^−^) human melanoma cells to determine if the new chelator had any effect on α_v_β_3_ binding in vitro. Results of these studies demonstrated that the [^89^Zr]Zr-FSC complex did not disrupt RGD-α_v_β_3_ binding in vitro, and this finding was corroborated using biodistribution and small animal PET/CT studies in nude mice bearing contralateral human melanoma M21 and M21L tumors. These studies revealed excellent retention of radioactivity in integrin positive tumors, and a complete biodistribution profile that was consistent with RGD-based radiopharmaceuticals [102]. In a recent publication, Summers, et al. extended the evaluation of the FSC ligand by conjugating it to the anti-EGFR affibody, ZEGFR:23377 using a maleimide-based bioconjugation strategy to produce FSC-ZEGFR:23377 [103]. This bioconjugate was radiolabeled in a facile manner using [^89^Zr]Zr(ox)_2_ and radiochemical methods typically used to prepare [^89^Zr]Zr–DFO-mAbs. Binding studies and biodistribution studies demonstrated that antigen reactivity was retained in vitro and in vivo, but at 24 h post-injection, the radioactivity level in the bone tissues of mice receiving the radiolabeled affibody was comparable to levels in the bones of mice receiving the [^89^Zr]Zr-DFO-bioconjugate. Clearly, significant progress has been made exploring FSC and its ^89^Zr radiochemistry, but additional studies to examine radioactivity levels in bone tissue at much later time points will be necessary to fully appreciate its potential as a ^89^Zr-chealtor.

The Boros group recently described the desferrichrome (DFC; **18**)-inspired ^89^Zr chelators **9**–**15** (Figure 4) [104]. Desferrichrome (DFC; **18**) is an ornithine-derived hexapeptidyl siderophore secreted by bacteria and fungi, but to ensure accessibility of the tris-hydroxamate coordinating groups during chelation, the naturally occurring ligand was reverse-engineered to be acyclic, and modified with the near infrared (NIR) dye, silicon rhodamine (SiR). Attaching the NIR dye allowed the researchers to monitor coordination kinetics during metal complexation, to identify the Zr-DFC complex during any subsequent purification steps and provide a multi-modal imaging platform to describe tissue residualization after in vivo injection. Radiochemical studies demonstrated that these ligands could be radiolabeled quantitatively at room temperature; while EDTA challenge studies, revealed that ^89^Zr[Zr]-**12** and [^89^Zr]Zr-DFO demonstrated comparable resistance to transchelation. Although biodistribution studies involving the radiometal chelates were not reported, **14**, which was an NCS-modified version of **12**, was conjugated to trastuzumab and radiolabeled with ^89^Zr in order to compare its stability to DFO when incorporated into a mAb-based radiopharmaceutical. Biodistribution studies conducted in normal C57Bl6 mice revealed accelerated blood clearance compared to [^89^Zr]Zr-DFO-trastuzumab, but animals injected with [^89^Zr]Zr-**14**-trastuzumab retained significantly more radioactivity in liver tissue, which may preclude the imaging of tumors within the abdominal cavity. Finally, radioactivity levels in bone tissue of mice receiving either [^89^Zr]Zr-**14**- or the [^89^Zr]Zr-DFO-trastuzumab were similar. Current experiments are underway to examine the NIR properties of **15** to determine if it can be applied in a multi-modal strategy that enables preclinical antibody development.

**Figure 5:**
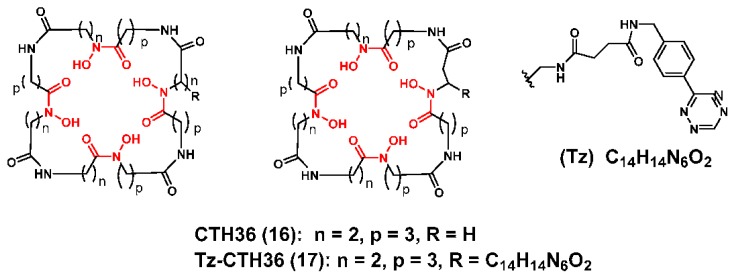
Macrocycle-based hydroxamate chelators for Zirconium-89. The coordinating units are depicted in red font.

Siebold and coworkers described the rational design and solid phase synthesis of CTH36 (**16**) as a ligand for ^89^Zr chelation (Figure 5) [105]. To maximize the potential of this new ligand its rational design was predicated on extensive computational studies and several important design characteristics including (1) the inclusion of four hydroxamate coordinating units; (2) a macrocyclic structure to take advantage of the macrocyclic effect; (3) rotational symmetry to limit isomers; (4) hydrophilic character; and (5) an optimal cavity size to provide a balance between ring strain and entropic effects. They also developed Tz-CTH36 (**17**) and conjugated it to a transcyclooctene-modified c(RGDfK) analog using and inverse electron demand Diels-Alder coupling strategy so that they could compare the radiochemistry and in vitro properties of this conjugate with [^89^Zr]Zr-DFO-c(RGDfK). Interestingly, both conjugates underwent facile radiolabeling, but [^89^Zr]Zr-CTH36-c(RGDfK) was more resistant to EDTA challenge. The authors postulated that the higher kinetic stability of [^89^Zr]Zr-CTH36-c(RGDfK) was a consequence of the rationally designed ligand and its ability to complex the ^89^Zr^4+^ ion in an octadentate manner. However, the authors did not report any in vivo data regarding the biodistribution of [^89^Zr]Zr-CTH36-c(RGDfK) or any data regarding the preparation and evaluation of a [^89^Zr]Zr-CTH36-mAb conjugate. Completion of these remaining studies will be crucial in validating this rational design approach and determining if **16** and its BFC version **17** will be useful in ^89^Zr-immuno-PET applications.

**Figure 6:**
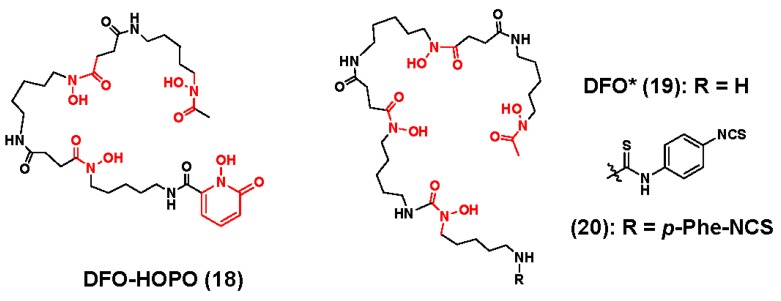
Octa-coordinate chelators for ^89^Zr inspired by DFO. The coordinating units are depicted in red font.

Another attempt to develop ligands that completely satisfy the ^89^Zr-coordination sphere was communicated by the Smith group, who evaluated the hybrid molecule DFO-1-hydroxy-2-pyridone (1**8**), which was originally designed for plutonium (IV) sequestration (Figure 6) [106]. Although nuclear magnetic resonance (NMR) analysis of ^Nat^Zr-**18** was not performed due its low solubility, the authors deduced a 1:1 metal-ligand complex using high resolution mass spectrometry. Radiochemical experiments demonstrated that the ligand could be quantitatively radiolabeled with excellent molar activity using comparable experimental conditions needed to prepare [^89^Zr]Zr-DFO, but unlike the latter, the former radiometal complex was inert to EDTA and serum challenge. Small animal PET/CT and acute biodistribution studies were also conducted on normal mice injected with either [^89^Zr]Zr-**18** or [^89^Zr]Zr-DFO. In contrast to the clearance profile of [^89^Zr]Zr-DFO, which underwent renal excretion exclusively, [^89^Zr]Zr-**18** demonstrated a bimodal excretion pattern. At early time points post-injection, rapid renal clearance was observed, but as the experimental time course progressed, hepatobiliary clearance predominated. The authors hypothesized that differences in hydrophilicity were responsible for the dichotomous excretion pattern of the former radiometal chelate. Moreover, radioactivity levels in the bone tissue of mice injected with [^89^Zr]Zr-**18** were significantly lower when compared to the radioactivity levels in the bone tissue of mice receiving [^89^Zr]Zr-DFO. Although current results demonstrate promise for this hybrid ligand, the BFC version must be evaluated before any conclusions can be made regarding its utility in ^89^Zr-immuno-PET applications.

Taking cues from Guerard et al. [98], Patra and coworkers, reported the DFO analog, DFO* (**19**), which was easily synthesized from the DFO-mesylate salt and the protected hydroxamic acid precursor (Figure 6) [107]. While derivatization to introduce functional groups for bioconjugation into **19** was facile, preparing and characterizing the ^Nat^Zr-**19** complex was challenging due to its poor solubility. Nevertheless, the authors were able to deduce a 1:1 metal-to-ligand binding motif using high resolution mass spectrometry and NRM analysis, but also noted the presence of structural isomers, which the authors attributed to the numerous coordination modes that the acyclic ligand can adopt during complexation of the Zr^4+^ ion. To understand how the addition of the fourth hydroxamate coordinating unit influenced the radiochemistry of this ligand, the authors prepared [^89^Zr]Zr-**19**-[NIe14]BBS(7–14) and [^89^Zr]Zr-DFO-[NIe14]BBS(7–14) with achievable molar activities of 5–6 GBq-µmol^−1^. Although no uncomplexed ^89^Zr was observed in the reaction mixture, radio-high performance liquid chromatography (HPLC) did detect the presence of isomers, which was consistent with the NMR solution data of the non-radioactive complex. When challenged with excess DFO, the [^89^Zr]Zr-**19** conjugate resisted transchelation. More importantly, LogD_7.4_ and in vitro binding studies using gastrin-releasing peptide receptor positive cell lines suggested that the addition of the fourth hydroxamate coordinating unit had minimal influence on the physical properties of the radiopharmaceuticals in vitro. Several years later, Vugts et al. extended this work by synthesizing **20**, which was prepared by reacting **19** with *p*-phenylenediisothiocyante, and then conjugating it to trastuzumab to yield a bioconjugate with an observed chelator-to-mAb ratio of 0.6–0.9 [108]. Additionally, comparative radiochemistry studies revealed that [^89^Zr]Zr-**D**FO*-*p*-Phe-NCS-trastuzumab and [^89^Zr]Zr-DFO-trastuzumab could be radiolabeled in high radiochemical yield within 60 min, but only [^89^Zr]Zr-DFO*-*p*-Phe-NCS-trastuzumab was more stable in a variety of storage media, and demonstrated a more robust immunoreactivity when challenged with the HER2/neu antigen. The authors attributed the superior in vitro stability of the DFO*-based radiopharmaceutical to the coordinatively saturated environment that the octa-coordinate ligand, **20** provides the ^89^Zr^4+^ ion. Biodistribution studies in nude mice bearing HER2/neu positive tumors derived from the human, N87 human gastric cancer cell line were also very descriptive. Although both radiopharmaceuticals had very similar blood clearance profiles, animals injected with the [^89^Zr]Zr-**D**FO*-*p*-Phe-NCS-trastuzumab demonstrated significantly lower radioactivity levels in liver, spleen, and bone tissue, which was corroborated by small animal PET/CT studies in the same xenograft model. The development of **20** represents a significant achievement in ^89^Zr chelator design, and since recent efforts to improve the water solubility of this ligand have been successful the radiopharmaceutical community anxiously awaits its evaluation in a clinical setting [109,110,111].

**Figure 7:**
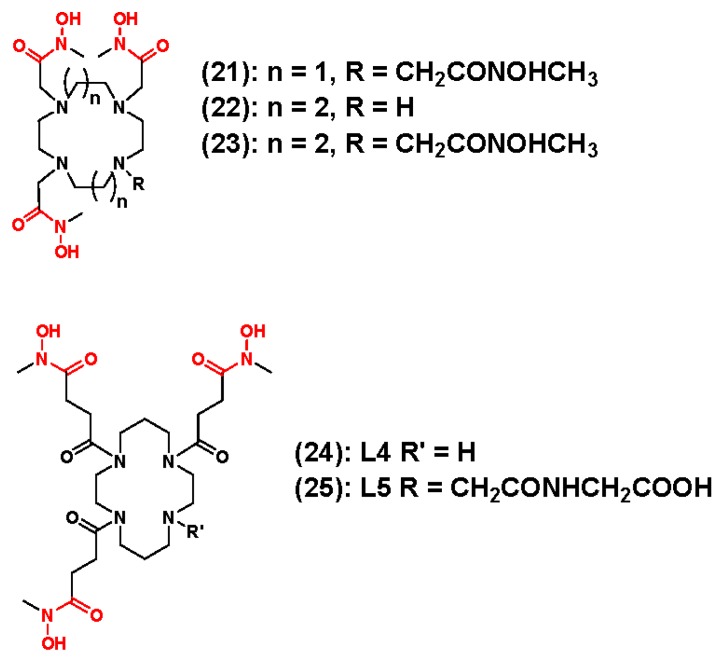
Cyclen-and cyclam-based ^89^Zr chelators containing hydroxamate pendant arms. The coordinating units are depicted in red font.

Additional hydroxamate-based ligands **21**–**24** were reported by Boros and coworkers [112]. These ligands are depicted in Figure 7. Creatively, the authors used the macrocycles cyclen and cyclam as molecular scaffolds, and alkylated the secondary amines within each macrocycle to yield chelators with three or four hydroxamte-functionalized pendant arms in relatively good overall yields. Further computational studies to optimize the length of the pendant arm generated a cyclam-based ligand (**25**) that underwent facile radiolabeling to produce an [^89^Zr]Zr-complex with improved stability over the cyclen-based chelators. EDTA challenge studies revealed [^89^Zr]Zr-**25** was 95% intact after 72 h, while LogD_7.4_ studies revealed a comparable hydrophilicity with ^89^Zr-DFO. Furthermore, biodistribution and small animal PET/CT studies in normal mice confirmed the in vitro results with rapid renal clearance of the administered radioactivity after only 30 min. Biodistribution data obtained 24 h post-injection revealed a biodistribution pattern similar to ^89^Zr-DFO, but to further assess **25** as a ^89^Zr chelator, the authors compared the in vivo pharmacokinetics of [^89^Zr]Zr-**25**-*N*-SUC-trastuzumab, which was prepared using a TFP-mediated approach, and [^89^Zr]Zr-DFO-trastuzumab using small animal PET/CT in a nude mouse model bearing bilateral BT474 (HER2+) and BT20 (HER2−) human breast cancer xenografts. As expected, efficient and specific targeting of the HER2/neu receptor was observed with HER2+ tumors easily visualized as image contrast improved over the experimental time course. However, image analysis and post-PET biodistribution results revealed that the radioactivity levels in the bones of animals injected with [^89^Zr]Zr-**25**-*N*-SUC-trastuzumab were 9-fold higher than that observed in the bones of mice receiving [^89^Zr]Zr-DFO-trastuzumab. This suggests that further optimization of the ligand structure will be necessary to improve ^89^Zr-chealte stability before these ligands are made available to the research community.

### 3.2. Zirconium-89 Chelators Containing Hydroxyisopthalamide, Terepthalamide and Hydroxypyridinone Coordinating Units

Over the last three decades, the prolific work by the Raymond group and others has led to the development of a vast library of rationally designed, pre-organized ligands containing multi-dentate catecholate, hydroxpyridinoate, hydroxyisopthalamide and terepthalamide coordinating units [86]. Although originally designed as decorporation agents for numerous radioactive ions such as plutonium (IV), uranium (IV) and thorium (IV), the radiochemistry community has recently rediscovered these ligands and made numerous attempts to adapt them for zirconium (IV) radiochemistry.

**Figure 8:**
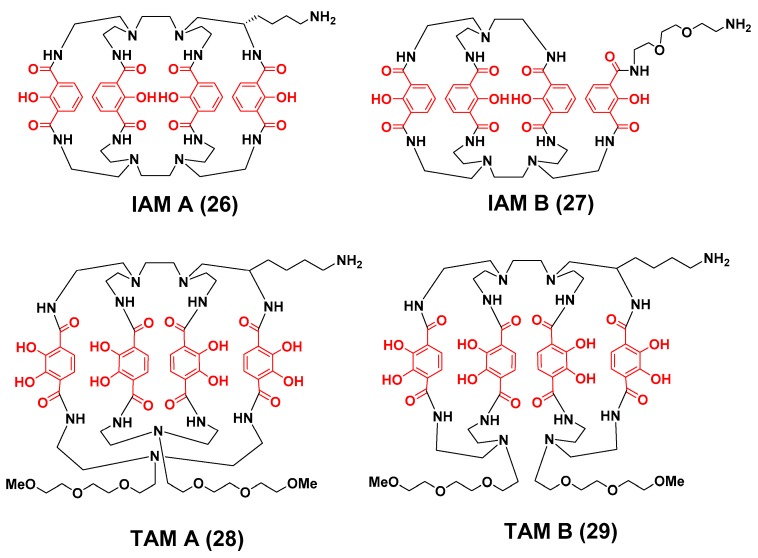
Bifunctional chelators for ^89^Zr containing hydroxyisopthalamide and terepthalamide coordinating units. The coordinating units are depicted in red font.

Bhatt et al. reported a pair of ligands containing hydroxyisopthalamide coordinating units, which were believed to bind ^89^Zr in an octadentate manner through a combination of phenolic and carbonyl oxygen atoms (Figure 8) [113]. Ligand **26** was developed as a rigid trimacrocycle comprised of 24 and 30 member rings whereas ligand **27** formed a more flexible bimacrocycle comprised of 24 and 27 member rings. The influence of these structural differences on in vitro and in vivo behavior was readily apparent. While both ligands could be quantitatively radiolabeled with ^89^Zr to yield high molar activity radiometal complexes, the more rigid **26** required forcing conditions. However, [^89^Zr]Zr-**26** was more resistant to DTPA and serum challenge, and demonstrated greater in vivo stability. For example, mice injected with [^89^Zr]Zr-**26** retained less activity in their liver, kidney and bone tissue than did those animals injected with [^89^Zr]Zr-**27**, but it still did not demonstrate pharmacokinetic properties that surpassed those of [^89^Zr]Zr-DFO.

Pandya et al. investigated the use of the 2,3-dihydroxyterepthalamide (TAM) coordinating units in ^89^Zr-BFC design [114]. These functional groups are highly acidic and exist as di-anions at neutral pH. The authors believed their incorporation into a macrocycle with an appropriate cavity size would yield an octadentate ligand with high avidity for the ^89^Zr^4+^ cation [114]. Moreover, Raymond and coworkers previously demonstrated how this coordination motif could be used to engineer the solution properties of the resultant chelating ligand [86]. Using high dilution conditions to avoid side reactions, the authors were able to isolate two distinct regioisomers. Regioisomer **28** was identified to have a more rigid structure containing two 29 atom macrocycles, while regioisomer **29** was identified as a more flexible “clam-shell” like system composed of two-26 atom ring systems. Despite the differences in rigidity, both ligands were quantitatively radiolabeled under the same conditions needed to prepare [^89^Zr]Zr-DFO, and neither radiometal complex demonstrated transchelation when challenged with excess DTPA or human serum. Interestingly, these complexes were more resistant to transmetallation than the IAM analogs and illustrate the greater affinity of the TAM coordination motif for the Zr^4+^ cation. Biodistribution studies conducted in normal mice revealed that the more rigid complex [^89^Zr]Zr-**28** was more stable in vivo than [^89^Zr]Zr-**29** and underwent more rapid clearance from all tissues over the experimental time course. The authors asserted that since **29** was a less rigid bi-macrocycle, the resulting ^89^Zr complex was more susceptible to perturbation by endogenous serum proteins and resulted in greater transchelation of the ^89^Zr^4+^ ion. The in vivo behavior of [^89^Zr]Zr-**28** was also compared with [^89^Zr]Zr-DFO. Although both radiometal complexes demonstrated a similar clearance pattern from the blood pool, [^89^Zr]Zr-**28** had significantly elevated levels of radioactivity in liver and kidney tissue, which the authors attributed to multifactorial processes including aggregation and changes in the molecular structure of [^89^Zr]Zr-**28** due to changes in intracellular pH within these tissues. Finally, levels of radioactivity observed in bone tissue of mice receiving [^89^Zr]Zr-**28** were comparable to that of mice injected with [^89^Zr]Zr-DFO. Typically, low incorporation of radioactivity in bone would stimulate further research into this ligand class, but the excessive retention of radioactivity in kidney tissue will most likely prohibit their further development as ^89^Zr chelators. 

**Figure 9:**
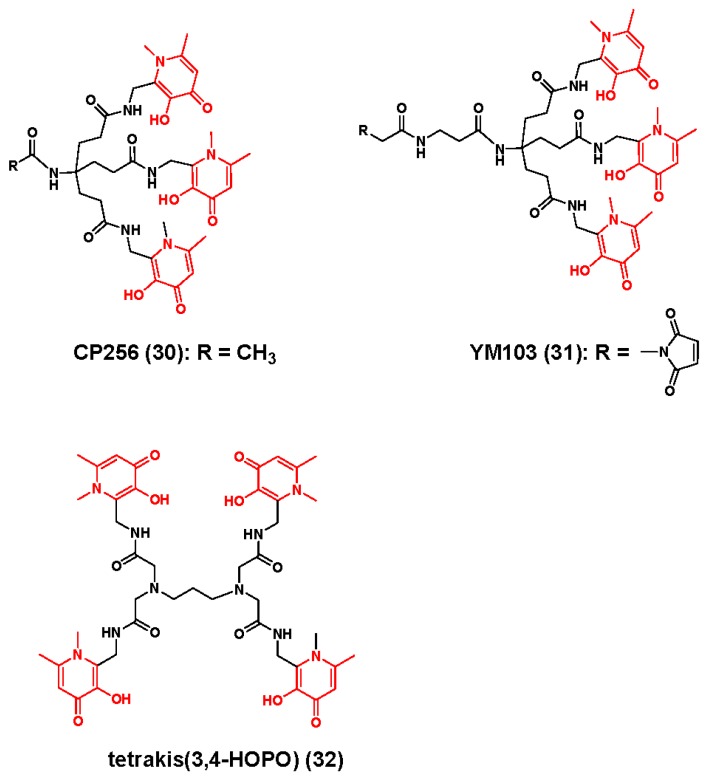
Zirconium-89 chelators containing 3,4-hydroxypyridinone coordinating units. The coordinating units are depicted in red font.

The Blower group reported CP256 (**30**) [115], which is based upon three 1,6,-dimethyl-3-hydroxy-pyridin-4-one groups, and is depicted in Figure 9. Although a single molecule crystal structure was not reported, high resolution mass spectrometry analysis in conjunction with ^1^H and ^13^C-NMR studies revealed that ^Nat^Zr-**30** formed with a 1:1 ligand-to-metal stoichiometric ratio. However a high degree of fluxionality was observed within the NMR spectra and suggested to the authors that multiple structural isomers existed in solution. Initial radiochemistry studies indicated that **30** could be quantitatively radiolabeled at a ligand concentration of 10 mM, but this became more difficult when the concentration of the latter approached the nanomolar range. Biodistribution studies in normal mice injected with [^89^Zr]Zr-**30** revealed a renal excretion pattern similar to that of [^89^Zr]Zr-DFO suggesting that [^89^Zr]Zr-**30** was stable on this rapid time scale. However, stability differences became more pronounced when the research team compared [^89^Zr]Zr-**31**-trastuzumab, which contained the bifunctional chelating version of **30**, and [^89^Zr]Zr-DFO-trastuzumab. At early biodistribution time points, similar levels of radioactivity were observed in the blood pool, spleen, liver, kidney and bone tissues regardless of the injected radiopharmaceutical. However, as the experimental study progressed, animals injected with [^89^Zr]Zr-DFO-trastuzumab seemed to retain less activity within tissue. For example, by the end of the study, the radioactivity associated with the bone tissue of animals receiving [^89^Zr]Zr-**31**-trastuzmab was 6-fold higher than that of animals receiving [^89^Zr]Zr-DFO-trastuzumab. Moreover, this accumulation of radioactivity in bone was clearly visible in small animal PET images, and forced the authors to conclude that **31** would not be a reliable ligand for the stable chelation of ^89^Zr where prolonged stability was required in vivo.

In an attempt to overcome the limitations of ligands **30** and **31**, Buchwalder and coworkers rationally designed 1,3,-propanediamine-*N*,*N*,*N*′,*N*′-tetrakis[(2-(aminoethy)-3-hydroxy-1-6-dimethyl-4(1*H*)-pyridinone)acetamide] (THPN, **32**), which is based upon four 3-hydroxy-4-pyridinone (3,4-HOPO) coordinating groups, in an attempt to fully satisfy the octadentate coordination sphere preferred by the ^89^Zr^4+^ ion [116]. Although traditional synthetic routes to this ligand proved laborious, a breakthrough was achieved when the research team adopted a microwave-assisted approach, which reduced the reaction time from six days to six hours and substantially increased the product yield. Computational studies and high resolution mass spectrometry of ^Nat^Zr-**32** revealed a 1:1 metal-to-ligand stoichiometry, while radiochemistry studies revealed radiolabeling kinetics that were superior to DFO in the micromolar range. LogP studies indicated that [^89^Zr]Zr-**32** was a highly hydrophilic complex and demonstrated a similar hydrophilicity to other [^89^Zr]Zr-HOPO and [^89^Zr]Zr-TAM complexes. Moreover, while serum stability and EDTA challenge studies at physiological pH revealed comparable stability between both radiotracers, studies under low pH conditions revealed that [^89^Zr]Zr-**32** was more resistant to demetallation suggesting it would remain stable in vivo. Finally, in comparative biodistribution and small animal PET/CT studies, the authors injected [^89^Zr]Zr-**32** or [^89^Zr]Zr-DFO into normal mice to evaluate the clearance of radioactivity from normal tissues. Image analysis revealed minimal amounts of radioactivity in tissue with a majority of the injected activity being excreted renally. A 24 h post-PET biodistribution analysis revealed a similar pharmacokinetic profile as [^89^Zr]Zr-DFO, and comparable radioactivity levels were observed in the bone tissues of both animal cohorts. Although the authors speculate that **32** could be a reasonable alternative to DFO, biodistribution and small animal PET imaging data describing the stability of this ligand as part of mAb-based radiopharmaceutical must be completed, and the radiopharmaceutical community anxiously awaits these results.

**Figure 10:**
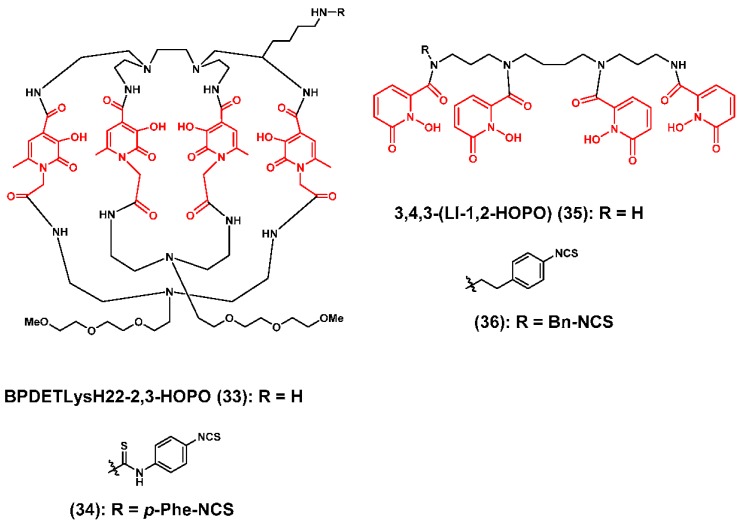
Zirconium-89 chelators containing 2,3-hydroxypyridinone and 1,2-hydroxypyridinone coordinating units. The coordinating units are depicted in red font.

A recent academic-industrial collaboration between Genentech, Inc., Lumiphore, Inc. and The Wake Forest School of Medicine resulted in the development of BPDETLysH22-2,3-HOPO (**33**), which contained four 3,2-HOPO coordinating units (Figure 10) [93]. The novel “clam shell” structure was envisioned to offer rapid ^89^Zr^4+^ complexation kinetics that would be similar to those of DFO, and improved radiometal complex stability because of the pre-organized macrocyclic design. Similar to ligands reported by Bhatt and Pandya [113,114], the synthesis of **33** involved the condensation of tetraamine and activated diacid intermediates under high dilution conditions to avoid oligomeric and polymeric by-products. While a 1:1 meta-to-ligand stoichiometric ratio was observed by high resolution mass spectrometry, NMR and HPLC analysis revealed the presence of structural isomers after ^Nat^Zr^4+^ ion coordination. Initial radiochemistry experiments using the conditions described for the preparation of [^89^Zr]Zr-DFO revealed that this ligand could be quantitatively radiolabeled with excellent molar activity in less than 30 min, and the resulting hydrophilic complex was more resistant to DTPA challenge than [^89^Zr]Zr-DFO. Biodistribution studies in normal mice were conducted with [^89^Zr]Zr-**33** and [^89^Zr]Zr-DFO to compare the clearance kinetics of each tracer, and revealed efficient blood clearance of both radiotracers over the 72 h experimental time course. However elevated levels of radioactivity were observed in the kidney, liver and bone tissues of mice receiving [^89^Zr]Zr-**33**. Next, the authors prepared the BFC version **34** and conjugated it to trastuzumab. They then compared [^89^Zr]Zr-2,3-HOPO-*p*-Phe-NCS-trastuzumab and [^89^Zr]Zr-DFO-trastuzumab in a HER2/neu mouse model of ovarian carcinoma using small animal PET/CT imaging. Time activity curves for tumor, blood, liver and bone were obtained over the 144 h experimental time course. As expected, radioactivity in the blood pool was comparable for each cohort regardless of the injected immuno-PET agent, and in vivo targeting of the HER2/neu tumor was efficient suggesting that the chelator did not alter trastuzumab specificity in vivo. However, animals receiving [^89^Zr]Zr-2,3-HOPO-*p*-Phe-NCS-trastuzumab did retain more radioactivity in liver and bone tissues when compared to animals injected with [^89^Zr]Zr-DFO-trastuzumab forcing the authors to conclude that further optimization of the macrocyclic scaffold would be necessary to improve radiometal complex stability and make these ligands viable DFO alternatives.

To date, the most successful application of the hydroxypyridinone coordinating unit in ^89^Zr chelator design was published by Deri and collaborators who described the acyclic 3,4,3-(Li-1,2-HOPO) ligand **35** [117], which comprised a linear spermine backbone appended with four 1,2-hydroxypyridinone coordinating units. The acyclic nature of the ligand, the low pKa values of the coordinating units and an ideal cavity size were believed to be important design characteristics that would facilitate ^89^Zr complexation and result in an ultra-stable radiometal complex. Similar to other ligands containing hydroxypyridinone coordinating units, ^89^Zr radiolabeling kinetics were rapid in the presence of excess ligand, but during these studies, the authors noted the presence of two distinct species, whose formation was concentration-dependent. Experimental evidence led the research team to hypothesize that the radiometal complex initially formed reflected a dimeric species involving two ligands binding one ^89^Zr^4+^ ion. However, over time, this dimeric complex interconverted to a second species, which the authors attributed to [^89^Zr]Zr-**35** containing the expected 1:1 metal-to-ligand stoichiometry. After isolating the later species, the authors evaluated its kinetic inertness, its resistance to transchelation and its selectivity for ^89^Zr^4+^ ion by challenging [^89^Zr]Zr-**35** with human serum, excess EDTA and biologically relevant metal ions, respectively. In human serum both [^89^Zr]Zr-**35** and [^89^Zr]Zr-DFO were more than 98% intact after 7 days at 37 °C, but when challenged with excess EDTA or biologically relevant metal cations, [^89^Zr]Zr-**35** was observed to be more resistant to demetallation. Biodistribution and small animal PET/CT studies revealed that in contrast to animals injected with [^89^Zr]Zr-DFO, animals injected with [^89^Zr]Zr-**35** displayed a biomodal excretion pattern with renal excretion occurring at early time points and hepatobiliary clearance occurring at later time points. Biodistribution studies indicated significantly more radioactivity in the bone tissue of mice injected with [^89^Zr]Zr-**35** than in the bones of mice receiving [^89^Zr]Zr-DFO, but the authors attributed these observations to differences in the perfusion and clearance kinetics exhibited by the two radiometal complexes rather than transchelation of the ^89^Zr into the phosphate rich bone matrix. 

In a subsequent publication, Deri and coworkers reported the bifunctional chelator analog of **35**, SCN-Bn-HOPO (**36**) [117]. Synthesis of this molecule was non-trivial as it required the incorporation of the *p*-benzylisothiocyanate pendant arm, which was used for antibody conjugation, into the symmetrical ligand. Although deprotection steps to achieve the final BFC proved challenging, it was eventually conjugated to trastuzumab yielding a bioconjugate with a 3:1 chealtor:mAb ratio. Satisfyingly, the ^89^Zr chelation kinetics of the BFC when attached to trastuzumab were similar to the initial ligand; to that observed with DFO-trastuzumab, and both conjugates were quantitatively radiolabeled with a specific activity of 74 MBq/mg in less than an hour. More importantly, both radiopharmaceuticals demonstrated immunoreactivities greater than 85% suggesting that the presence of the [^89^Zr]Zr-HOPO complex on the antibody surface did not affect in vitro stability or HER2/neu receptor affinity. The in vivo performance of both radiopharmaceuticals was compared using small animal PET/CT imaging and acute biodistribution studies in a murine model of HER2/neu positive breast cancer. Both agents demonstrated efficient tumor accumulation with improving image contrast as they cleared from non-target tissues. However, mice injected with the HOPO-based radiopharmaceutical demonstrated reduced accumulation of radioactivity in their skeleton over the experimental time course of this study. Biodistribution data corroborated the imaging results and revealed that the radioactivity level in the skeleton of mice receiving [^89^Zr]Zr-1,2-HOPO-Bn-SCN-trastuzumab was approximately 8-fold lower than for mice receiving the [^89^Zr]Zr-DFO conjugate. Accordingly, the former radiopharmaceutical afforded a better tumor-to-bone ratio, which may be an important criterion in reducing false positive rates associated with ^89^Zr-immuno-PET-based bone metastasis detection strategies [97,118]. Additionally, the improved contrast may enable more accurate biodistribution and dosimetry in advance of targeted systemic radiotherapy, and the successful use of this ligand in immuno-PET applications stands out as another excellent success story in the preclinical development of ^89^Zr chelators.

### 3.3. ^89^Zr Chelators Containing Tetraazamacrocycles

**Figure 11:**
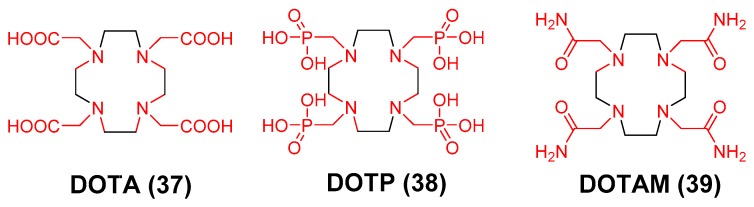
Zirconium-89 chelators containing tetraazamacrocycles. The coordinating units are depicted in red font.

Polyaminocarboxylate ligands, which include acyclic ligands such as ethylenediamine- tetraacetic acid (EDTA), diethylenetriaminepentaacetic acid (DTPA) or tetraazamacrocycles such as 1,4,7,10-tetraazacyclododecane-1,4,7,10-tetraacetic acid (DOTA, **37**) represent one ligand class that has been at the forefront of radiopharmaceutical development for nearly half a century (Figure 11) [14,15]. While useful for stably chelating a variety of radiometals, reports describing them as ^89^Zr chelators are absent in the literature despite a strong preference of Zr(IV) for polyanionic hard donor ligands and the very impressive stability constants of some of the resulting Zr-complexes. Recently, Pandya et al. reported their initial observations on ^Nat/89^Zr-tetraazamacrocycle complexes by preparing Zr-**37**, Zr-**38** and Zr-**39** and characterizing each complex using high resolution mass spectrometry, and ^1^H- and ^13^C-NMR [119]. Fortunately, the molecular structure of Zr-**37** was elucidated by single crystal x-ray diffraction analysis. It provided irrefutable proof of an octadentate coordination environment where all four macrocycle nitrogen atoms and acetate pendant arms participated in Zr^4+^ ion coordination. Furthermore, the compressed, square anti-prismatic geometry and low-symmetry saddle-like ligand conformation displayed by Zr-**37** was consistent with the limited number of reports describing Zr-catalysts containing analogous ligands with a Zr(IV) metal center that lacks crystal-field stabilization [120,121,122,123,124,125,126,127,128].

After completing synthesis and characterization of the reference complexes, attempts to prepare the radioactive analogs using standard procedures published for the preparation of [^89^Zr]Zr-DFO resulted in low radiochemical yields. However, the authors seized upon earlier work by Holland et al., who described the use of [^89^Zr]ZrCl_4_ as potential alternative to [^89^Zr]Zr(ox)_2_ in ^89^Zr-immuno-PET synthesis [75]. Using this radioactive precursor, they were able to quantitatively prepare [^89^Zr]Zr-**37**, [^89^Zr]Zr-**38**, and [^89^Zr]Zr-**39** with specific activities that were similar to other ^89^Zr-complexes published in the literature. To explain differences in ligand reactivity, the authors rationalized the ^Nat/89^Zr species present in solution dictated complex formation [63]. Zirconium oxalate is a highly stable complex, even under highly acidic conditions and at very low molar concentrations. Accordingly, when [^89^Zr]Zr(ox)_2_ is reacted with the tetraazamacrocycle, the oxalate anion’s ability to form a stable complex in aqueous media effectively competes with the macrocycle, resulting in a low radiochemical yield of the [^89^Zr]Zr-tetraazamacrocycle complex. Conversely, ZrCl_4_, is highly charged, oxophilic and readily undergoes aquation in solution to form multiple µ-hydroxo-and µ-oxo-bridged species. Thus, in the absence of a competing oxalate ligand, [^89^Zr]Zr-tetraazamacrocycle complex formation was favored when [^89^Zr]ZrCl_4_ was reacted with the macro-cycle in solution [63]. 

In vitro, [^89^Zr]Zr-tetrazamacrocycle complexes demonstrated remarkable stability with [^89^Zr]Zr-**37** being the most inert to serum, EDTA or biologically relevant metal ion challenge. Acute biodistribution studies were also conducted to examine stability in vivo. Mice intravenously injected with [^89^Zr]Zr-**37** retained significantly less radioactivity in their tissues compared to mice injected with [^89^Zr]Zr-**38** or [^89^Zr]Zr-**39**. More importantly, biodistribution and small animal PET/CT studies demonstrated that mice injected with [^89^Zr]Zr-**37** retained less radioactivity in their tissues than did animals injected with [^89^Zr]Zr-DFO suggesting the former is superior to the latter in terms of in vivo stability. Although results describing **37** as part of an ^89^Zr-immuno-PET agent are still unreported; if successful, use of tetraazamacrocycles in ^89^Zr-immuno-PET may pave the way for truly theranostic approach to targeted systemic radiotherapy since it would be possible to radiolabel one FDA approved DOTA-mAb conjugate with ^89^Zr and therapeutic radionuclides [70,129,130,131,132,133,134,135,136,137,138,139,140,141,142]. This strategy may increase dosimetric accuracy, reduce regulatory burden, and minimize costs associated with cGMP-compliant radiopharmaceutical development, so that they may be more readily integrated into personalized medicine strategies in the future. 

## 4. Conclusions

Zirconium-89 radiopharmaceutical research has progressed rapidly since this radionuclide was first produced more than three decades ago; great strides have been made to standardize its production, understand aqueous zirconium chemistry, and design ligands that stably chelate this important PET isotope. As a consequence, numerous siderophore-inspired ligands containing hydroxamate; hydroxyisopthalamide; terepthalamide, and hydroxypiridinoate coordinating units have been scrutinized and reported to effectively chelate ^89^Zr. Thus far, only DFO* and HOPO derivatives have proven effective as ^89^Zr-chelators when incorporated into an antibody-based radiopharmaceutical, while others chelators still await evaluation in this context. Interest in tetraazamacrocycles as ^89^Zr chelators has also increased given the recent revelation that this ligand class can form ultra-stable ^89^Zr complexes. Undoubtedly, the next few years will see this highly dynamic research area yield new insights and exciting breakthroughs in ^89^Zr-immuno-PET radiopharmaceutical design that will have a transformative impact on how precision medicine strategies are implemented in the clinic.

## Abbreviations

BFC	bifunctional chelator
Bq	bequerel
Ci	curie
DFC	desferrichrome
DFO	desferrioxamine B
DOTA	1,4,7,10-tetraazacyclododecane-1,4,7,10-tetraacetic acid
DTPA	diethylenetriaminepentaacetic acid
EDTA	ethylenediaminetetraacetic acid
FSC	fusarine C
GBq	gigabequerel
HOPO	hydroxpyridinoate
IAM	hydroxyisopthalamide
kBq	kilobequerel
keV	kiloelectron volt
MBq	Megabequerel
mCi	milicurie
MeV	megaelectron volt
PET	positron emission tomography
TAM	terepthalamide
TFAC	triacetylfusarine C
THPN	1,3-propanediamine-*N*,*N*,*N*′,*N*′-tetrakis[(2-(aminoethy)-3-hydroxy-1-6-dimethyl-4(1*H*)-pyridinone)acetamide]
uCi	microcurie
α++	alpha particle
β−	beta minus particle
β+	positron
γ	gamma photon
